# Cell-Assisted Lipotransfer: A Systematic Review of Its Efficacy

**DOI:** 10.1007/s00266-016-0613-1

**Published:** 2016-02-18

**Authors:** Navid Mohamadpour Toyserkani, Marlene Louise Quaade, Jens Ahm Sørensen

**Affiliations:** Department of Plastic and Reconstructive Surgery, Odense University Hospital, Odense C, Denmark; Department of Clinical Biochemistry and Pharmacology, Odense University Hospital, Odense C, Denmark

**Keywords:** Regenerative medicine, Stromal vascular fraction, Adipose-derived stromal cell, Cell-assisted lipotransfer, Fat graft

## Abstract

**Introduction:**

Autologous lipotransfer is seen as an ideal filler for soft tissue reconstruction. The main limitation of this procedure is the unpredictable resorption and volume loss of the fat graft. In the recent decade, an increasing amount of research has focused on the use of adipose tissue-derived stromal cells (ASCs) to enrich the fat graft, a procedure termed cell-assisted lipotransfer (CAL). The aim of this review was to systematically review the current preclinical and clinical evidence for the efficacy of CAL compared with conventional lipotransfer.

**Materials and Methods:**

A systematic search was performed on PubMed and other databases to identify all preclinical and clinical studies where CAL with ASCs was compared with conventional lipotransfer. A total of 20 preclinical studies and seven clinical studies were included in the review.

**Results:**

The preclinical studies consisted of 15 studies using immunodeficient animal models and five studies using immunocompetent studies. Seventeen studies examined weight/volume retention of which 15 studies favored CAL over conventional lipotransfer. One clinical study did not find any efficacy of CAL and the remaining six studies favored CAL.

**Conclusions:**

The present evidence suggests that there is a big potential for CAL in reconstructive surgery; however, the present studies are so far still of low quality with inherent weaknesses. Several aspects regarding CAL still remain unknown such as the optimal degree of cell enrichment and also its safety. Further high-quality studies are needed to establish if CAL can live up to its potential.

**Level of Evidence V:**

This journal requires that authors assign a level of evidence to each article. For a full description of these Evidence-Based Medicine ratings, please refer to the Table of Contents or the online Instructions to Authors www.springer.com/00266.

## Introduction

Autologous fat transplantation for soft tissue augmentation to reconstruct congenital or acquired tissue defects is an increasingly ideal method in the field of plastic surgery. Autologous fat transplantation was first described over a century ago and is now seen as an ideal filler because it is host compatible, readily available, and can be harvested easily and repeatedly as needed without complications arising from allergic or foreign body reactions. Although adipose transfer has been widely used in clinical cases, high variability in long-term results is being reported, mainly due to unpredictable degrees of resorption and volume loss with graft survival rate ranging from 20 to 90 % in the present literature [1–5]. The low survival rate and high reabsorption rate of the transplanted fat reduce the efficacy of this technique. Many have tried to standardize different steps involved with lipotransfer but the survival rate is still very variable.

Because of the unpredictability associated with lipotransfer, researchers have sought to increase the survival rate of the transplanted fat by addition of growth factors such as insulin, vascular endothelial growth factor (VEGF), and many others [6–10] but also by enriching the fat with adipose tissue-derived stromal cells (ASCs) [11].

The number of ASCs in adipose tissue is so pronounced that they can be harvested, isolated, and used for treatment as an autologous therapy in a single-stage procedure, which circumvents many ethical and immunologic questions associated with allogeneic/xenogeneic therapy. The stromal vascular fraction (SVF) is obtained from lipoaspirate, and consists of a heterogeneous group of cells including hematopoietic cells, endothelial cells, pericytes, and stromal cells [12, 13]. When these cells are cultured only the plastic adherent cells remain. This cell population is termed ASCs and they are more homologous in their phenotype than SVF cells and resemble mesenchymal stem cells obtained from the bone marrow [14].

In vivo models examining the use of SVF or ASCs for enrichment of fat grafts were first introduced in 2006 by Moseley et al. [15] and Matsumoto et al. [11] based on previous work by Llull and other unpublished work. Matsumoto et al. [11] first introduced the term cell-assisted lipotransfer (CAL) and since then many others have examined the efficacy of this technique with slight variations and promising results [16–20]. In recent years, the first human studies examining the efficacy of CAL have also been published [21–23].

The aim of this article was to systematically identify and review the current preclinical and clinical studies regarding CAL to see what lessons can be learned so far and what the current status of this technique is for clinical use.

## Adipose-Derived Stromal Cells

The adipose tissue plays important physiological roles in the human body as an endocrine organ and a site of energy storage. It is also utilized surgically as a filler in soft tissue augmentation for the correction of tissue defects or cosmetic purposes. Additionally, adipose tissue is now recognized as a rich source of stromal stem cells, which has opened for cell-based therapies in tissue remodeling [24–26]. The advantages of this source of stem cells are that it is easy accessible and that the harvesting procedure is minimally invasive compared to conventional stem cell sources like the bone marrow. Additionally, the yield of stem cells when harvesting from adipose tissue is estimated to be about 500-fold higher than that of bone marrow [27].

The adipose tissue is harvested by liposuction and the lipoaspirate is subsequently enzymatically digested by collagenase and fractionated into the SVF. This heterogeneous population is composed of many types of cells among these the stromal stem cells. The following surface markers identify the stromal population of the SVF phenotypically: CD13+ CD73+ CD90+ CD34+ CD31− CD45− [2]. When cultured the cell population becomes more homogeneous as non-adherent cells are eliminated and viable cells proliferate. The resulting population of ASCs have positive markers in common with other mesenchymal stromal/stem cells (MSCs), including CD105, CD44, CD73, CD90, CD13, and CD29, and negative for CD31 and CD45. The marker CD34 is generally expressed in a lower degree by ASCs but is dependent on culture conditions and the marker tends to diminish with increasing passages [28, 29]. Additionally, the ASCs are positive for CD36 and negative for CD106 distinguishing them from bone marrow-derived MSCs [14].

The SVF and ASC populations are multipotent and are therefore able to differentiate along several lineages (ectodermal [30], endodermal [31] and mesenchymal [32, 33]) to terminally differentiated cells. Additionally, the cells are able to secrete a series of growth factors, immune modulatory factors, and cytokines [34].

**Fig. 1:**
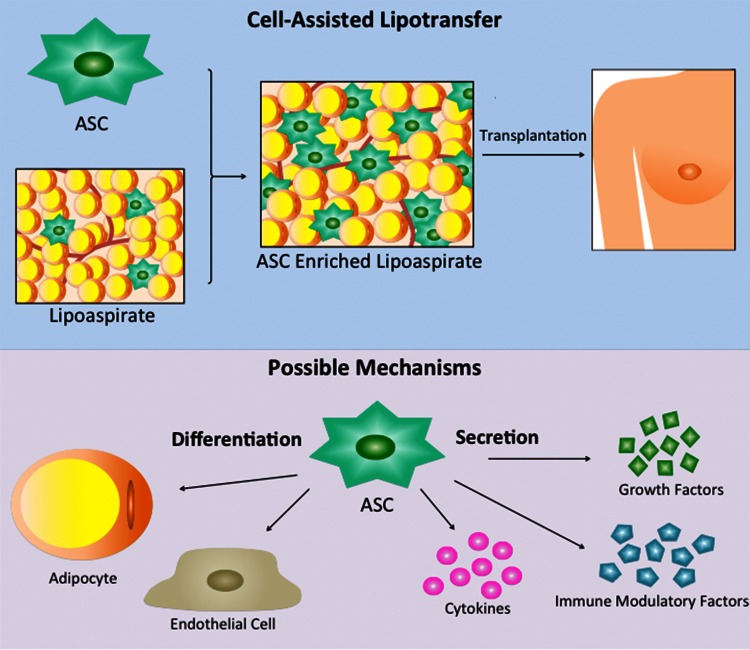
Schematic representation of the principle of cell-assisted lipotransfer and the possible mechanisms of action of the ASCs

As we report herein, many studies have showed promising results using SVF or ASCs in CAL. Several possible mechanisms for this positive effect on fat graft survival have been suggested (Fig. 1) [35]. Firstly, the multipotent capacity of the cells allows them to differentiate into cell types such as adipocytes or endothelial cells [15, 36], in this way contributing to the regeneration of the adipose tissue or promotion of angiogenesis, respectively. Secondly, ASCs may remain as original ASCs in the graft [11]. Finally, the mechanism could be a paracrine kind. ASCs secrete a wide range of factors that may influence the surrounding host tissue. An example is the growth factors HGF and SDF-1 that are released in response to injury and promote angiogenesis [37–39]. Regardless of the underlying mechanism, lipoaspirate enriched with SVF or ASCs seems to affect the commonly occurring postoperative atrophy and replacement process of grafted fat [8], making CAL a promising treatment.

## Data Acquisition

**Fig. 2:**
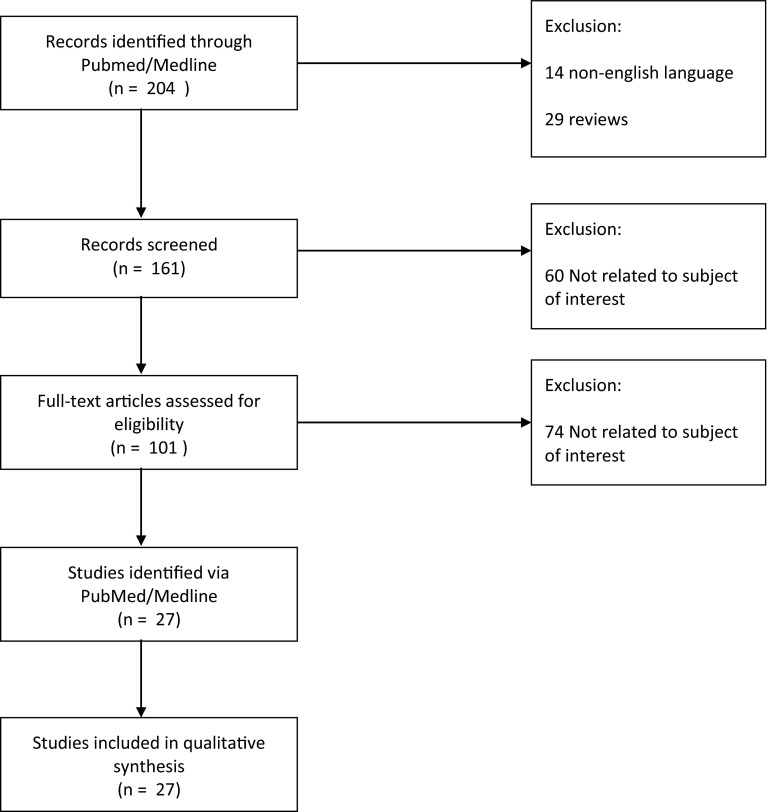
Flow diagram of search strategy

A systematic search was performed on PubMed from January 2000 to March 2015 to identify all animal and human studies in which CAL with ASCs or SVF had been compared with regular lipotransfer. The following search strategy was used “[(adipose stem cell) OR (adipose stromal cell) OR (adipose regenerative cell) OR (SVF)] AND [(fat graft) OR (lipotransfer) OR (lipofilling)].” The search was performed independently in duplicate by authors NMT and JAS. This search resulted in 204 studies. Review articles (29) and non-English language articles (14) were excluded leaving 161 articles. Inclusion criteria were any animal or human study, in which CAL was compared with non-CAL. All abstracts of remaining papers were skimmed and full text copies of relevant papers were acquired (60 not relevant based on abstract). From the remaining papers, 27 were included for analysis. See Fig. 2 for schematic overview of the search process. Reference lists of included papers were checked for additional studies without further findings. A similar search was performed on EmBase.

All included preclinical studies were checked for at least one outcome variable regarding lipotransfer retention rate using weight or volume measurements, histologic assessment of graft morphology using standardized scoring that was explained or capillary density with explanation for how the measurement was performed. All included clinical studies were checked for at least one radiologic outcome variable regarding lipotransfer retention [computed tomography (CT) or magnetic resonance imaging (MRI)]. Data adhering to these principles were included in this review, and if not, they were excluded.

## Results

### Animal Studies

#### Animal Models

Fifteen studies were identified in which immunodeficient animals were used. One study used rats [40] and the remaining studies used mice [11, 15, 18–20, 40–49]. Dosage for cell enrichment ranged from 5 × 10^5^ to 2 × 10^7^ cells/ml of lipoaspirate. In all studies, human tissue and cells were used [11, 18–20, 40–47] except for three in which rat [49] or mice [15, 48] tissue and cells were used. The relative survival improvement for simple CAL ranged from 1.19- to 2.37-fold compared with their respective control group, which consisted of regular lipotransfer.

Two studies found no significant difference in survival rate of which one had used SVF [40] and the other ASCs [42]. One study did not measure survival rate [41]. Four studies systematically examined the morphology of the explanted fat grafts of which two found significantly better integrity and less cyst formation [40, 42, 43, 49]. Many of the remaining studies described better morphology with more viable adipocytes, less necrosis, and cyst formation with CAL but they did not provide either sufficient description of their method or statistics to substantiate these claims. Ten studies examined capillary density of which 9 found a significantly increased capillary density with CAL. See Table 1.

**Table 1:** Overview of all preclinical studies using immunodeficient animals

	Donor	Recipient	Cell type	Location	Dosage/ml fat	Relative survival rate	Weight volume	Morphology	Vessel density
Ko et al. [42]	Human	Mice	ASC	Skull	1 × 10^6^–1 × 10^7^	n.s	n.s	n.s	0
Matsumoto et al. [11]	Human	Mice	SVF	Dorsum	d.u.	1.37	↑	0	0
Xu et al. [45]	Human	Mice	ASC	Dorsum	2 × 10^7^	2.37	↑	0	↑
Li et al. [20]	Human	Mice	SVF	Dorsum	2.5 × 10^6^	2.18	↑	0	↑
Li et al. [43]	Human	Mice	ASC	Dorsum	1 × 10^4^–1 × 10^7^	1.46	↑	↑	↑
Luo et al. [19]	Human	Mice	ASC	Dorsum	3.3 × 10^6^	1.28	↑	0	↑
Lu et al. [18]	Human	Mice	ASC	Dorsum	1.67 × 10^7^	2.22	↑	0	↑
Garza et al. [41]	Human	Mice	ASC	Dorsum	1 × 10^6^	0	0	0	↑
Jiang et al. [47]	Human	Mice	ASC	Dorsum	5 × 10^5^	1.48	↑	0	↑
Conde-Green et al. [40]	Human	Rat	SVF	Dorsum	6.67 × 10^6^	n.s	n.s	n.s	n.s
Zhou et al. [49]	Rat	Mice	SVF	Dorsum	d.u.	1.56	↑	↑	↑
Moseley [15]	Mice	Mice	SVF/ASC	Skull	d.u.	2.5 SVF ASC n.s	↑	0	0
Fu et al. [48]	Mice	Mice	SVF	Skull	5 × 10^5^	1.19	↑	0	0
Zhu et al. [46]	Human	Mice	SVF	Dorsum	2 × 10^6^	1.19	↑	0	0
Luo et al. [44]	Human	Mice	ASC	Dorsum	1–8 × 10^6^	0	↑	0	↑

*d.u* dose unknown, *n.s.* not significant, *0* not measured, ↑ improved/increased

Five studies were identified, in which immunocompetent animals were used of which two used rodents (allogeneic cells) [16, 50] and the remaining studies used rabbits (autologous cells) [17, 51, 52]. The relative survival improvement for CAL ranged from 1.29- to 4.14-fold compared with their respective control group. One study did not measure survival rate [17]. Three out of the four studies that systematically examined the morphology of the explanted fat grafts showed a significantly better integrity and less cyst formation with CAL. Two studies examined the capillary density and both found a significantly increased capillary density with CAL. See Table 2.

**Table 2:** Overview of all preclinical studies using immunocompetent animals

	Type of transplantation	Recipient	Cell type	Location	Cell number/ml fat	Relative survival rate	Weight volume	Morphology	Vessel density
Zhu et al. [50]	Allogenic	Mice	SVF	Skull	8.33 × 10^7^	2.06	↑	↑	↑
He et al. [51]	Autologous	Rabbit	SVF	Dorsum	d.u.	2.13	↑	n.s	0
Ni et al. [52]	Autologous	Rabbit	SVF	Dorsum	d.u.	1.29	↑	0	0
Seyhan et al. [16]	Allogeneic	Rat	ASC	Skull	d.u.	4.14	↑	↑	0
Piccinno et al. [17]	Autologous	Rabbit	ASC	Upper lip	5 × 10^6^	0	0	↑	↑

*d.u* dose unknown, *n.s.* not significant, *0* not measured, *↑* improved/increased

In summary of all preclinical studies, a majority of studies that measured survival rate found a significant increase in survival rate when using CAL (16 out of 18 studies). Although many studies noticed an improved morphology with CAL only 8 studies examined the morphology systematically of which 5 studies showed an improvement in morphology. Capillary density was examined in the majority of studies and 11 out of 12 studies found an increased capillary density.

#### Number of Administered ASCs

Four of the included studies used different dosages of cells. This is a very important area because the number of cells needed for the optimal augmented effect of CAL is not known. Ko et al. compared the use of 1 × 10^6^ and 1 × 10^7^ ASCs in a nude mouse model using human cryopreserved lipoaspirates and cells transplanted to the scalp [42]. Volumetric and weight analysis was performed at both 4 and 15 weeks postoperatively, and whereas the graft retention at 4 weeks marginally favored the higher dose when looking at weight but not volume there was no difference at all at 15 weeks between any of the groups with control. This study should be interpreted with caution as the viability of the cryopreserved lipoaspirates and cells was not evaluated.

Luo et al. compared four different dosages of human ASCs that were sorted for CD31 before being cultured in endothelial growth medium [44]. The dosages ranged from 1 to 8 × 10^6^ ASCs/ml and were injected into the dorsum of immunodeficient mice (0.5 ml of lipoaspirate per injection). They found that the highest dose of 8 × 10^6^ cells yielded the best retention rate, which was significantly better than all other groups.

Ni et al. performed a study that compared four different fat tissue to SVF ratios in an immunocompetent rabbit model [52]. They used a total of 0.8 g with varying ratios between fat and SVF. The ratios were 1/3, 1/4, 1/5, and 1/6 SVF. Although all SVF groups survived significantly better than the control group, the ratio of 1/4 was significantly better than the lower ratios. The ratios of 1/3 and 1/4 were very similar. These two studies suggest that there could be a lower threshold for the amount of cells needed for a beneficial effect.

Li et al. compared four different ASC concentrations ranging from 1 × 10^4^–1 × 10^7^ ASCs/ml of fat [43]. All fat grafts were supplemented with the same amount of platelet-rich plasma (PRP). At a follow-up of 90 days they found a significantly improved survival rate of transplanted fat using 1 × 10^5^ ASCs compared with the other groups which was complemented by an increased capillary density and also increased normal adipose tissue structure. This study suggests a possible upper limit of the amount of cells to enrich the fat in order to obtain optimal results. This is a somewhat surprising result as 1 ml of fat is considered to have more than 1 × 10^5^ of SVF with a normal range of 2–7 × 10^5^ per ml of fat [53–55].

#### The Effect of Culturing ASCs

Several authors have tried to compare the efficacy of SVF and ASCs using many different animal models. Bai et al. [56] could not demonstrate any difference in effect in a myocardial infarction model, whereas Harada et al. [57] and Sermon et al. [58] both showed an edge toward SVF in an ischemic limb and experimental autoimmune encephalomyelitis model, respectively. Only the study by Moseley et al. [15] compared the use of SVF and ASCs but this study did not describe the methodology or results in detail. A relative survival rate improvement of 2.5 was seen in the SVF group compared with control, whereas the ASCs group only showed a trend toward improvement but the difference was not significant.

In total, eleven studies used ASCs for CAL of which two did not evaluate survival rate. Two out of the remaining nine studies failed to demonstrate any difference from the control group. In total, ten studies used SVF of which one did not evaluate survival rate. One out of the remaining eight studies did not differ from the control group. With the present evidence available not much can be concluded regarding which cell population (fresh or cultured) is optimal.

#### The Effect of ASCs Modification and/or Supplementation

Several studies have examined if the benefit of CAL can be augmented with either genetic modification or supplemented with certain growth factors. All studies in this field suggest that CAL can be augmented with either modification or supplementation.

PRP has been used in several studies as an adjunct to stem cell treatment and this is also true for CAL. Seyhan et al. compared the use of CAL with ASCs with or without PRP or lipotransfer with PRP alone [16]. They found that CAL + PRP resulted in the best retention rate and the relative improvement was 1.4 when comparing with CAL alone.

VEGF stimulates angiogenesis and is secreted by ASCs and the paracrine effect of these cells is due partly to the secretion of this growth factor. Studies have either genetically modified cells to overexpress VEGF [18] or supplemented the cells with VEGF polylactic acid nano-sustained release microspheres for a sustained additional release of VEGF independent of the cells [20]. Both these studies showed an additional benefit of VEGF with a relative improvement in survival rate of 1.23 and 1.15, respectively, compared with regular CAL. This was statistically significant in both studies.

Luo et al. examined if supplementing CAL with 17-β Estradiol (E2) would yield greater survival rate [19]. A survival rate of 76.9 + 1.9 versus 71.2 + 1.7 % was seen for CAL + E2 and CAL only, respectively, which was statistically significant. Their histologic examination revealed a greater capillary density in the CAL + E2 group.

Jiang et al. found that supplementing CAL with basic fibroblast growth factor (bFGF) resulted in both higher survival rate of 81 + 5 versus 71 + 7 % and a higher vessel density when compared with the regular CAL group [47].

### Human Studies

#### Non-randomized Studies

Although many case series with CAL have been published since Yoshimura first presented the technique for human use not many have done so in a proper clinical trial setting [59]. Several authors have published case series using CAL but have not compared it with regular lipotransfer [22, 60]. A total of seven human studies were found (two randomized clinical trials) where CAL was compared with regular lipotransfer and outcome was assessed with either CT or MRI. In all studies except one, an increase in relative survival rate of CAL compared with regular lipotransfer is seen. See Table 3 for an overview of human studies.

**Table 3:** Overview of all human studies comparing cell-assisted lipotransfer with regular lipotransfer

	Study design	Recipient site	Cell type	Radiologic method	Relative survival rate
Li et al. [61]	Cohort study	Face	SVF	CT	1.4
Gentile et al. [62]	Cohort study	Face	SVF	MRI	1.6
Gentile et al. [63]	Cohort study	Breast	SVF	MRI	1.6
Koh et al. [65]	Randomized clinical trial	Face	ASC	CT	1.5
Peltoniemi et al. [23]	Cohort study	Breast	SVF	MRI	n.s
Chang et al. [64]	Cohort study	Face	SVF	CT	1.17
Kølle et al. [66]	Randomized clinical trial	Arm	ASC	MRI	5.0

*n.s.* not significant

Li et al. conducted a trial for patients undergoing lipotransfer for facial contouring where 26 patients underwent CAL with SVF and 12 patients underwent non-CAL based on a voluntary principle [61]. Patients underwent CT scans and had photographs taken before the procedure and 6 months after. For the CAL group, approximately the same amount of lipoaspirate was used for SVF isolation as was injected into the face. As determined by CT images they found a significantly better survival rate in the CAL group, which was 64.8 + 10.2 versus 46.4 + 9.3 % (*p* < 0.01).

Gentile et al. conducted a trial for patients undergoing lipotransfer because of facial scarring for various reasons [62]. They included 30 patients in three groups with 10 patients in each group. The groups were CAL with SVF, lipotransfer with PRP, and regular lipotransfer. Results were evaluated with both MRI and ultrasonography (US). A survival rate of 63 % was found for CAL versus 39 % in the control group which was statistically significant. This study did not describe their allocation algorithm or if any blinding was performed. The amount of lipoaspirate used for SVF isolation as well as the amount of fat transferred in each group was not described.

Gentile et al. conducted a trial for patients undergoing breast reconstruction with lipotransfer [63]. They included 10 patients that received CAL with SVF, 13 patients that received lipotransfer + PRP, and a control group of 10 patients with regular lipotransfer. Results were evaluated by MRI. They used approximately half of the lipoaspirate for SVF isolation which was then mixed with the remaining lipoaspirate before injection. Survival rate after 1 year was 63 % for the CAL versus 39 % in the control group which was statistically significant. This study did not describe their allocation algorithm or if any blinding was performed.

Peltoniemi et al. have compared SVF-enriched lipotransfer with regular lipotransfer for breast augmentation using the water-assisted liposuction technique in 18 patients [23]. Patients were offered to pay additional money for the SVF enrichment and as such the study was not randomized or blinded. They did not count the amount of SVF cells used for enrichment but the ratio between fat used for SVF isolation and the fat graft itself was approximately 1:1. The survival rate of CAL was 74 versus 79 % in the regular lipotransfer group measured using MRI, which was not statistically significant.

Chang et al. compared SVF-enriched lipotransfer with regular lipotransfer in patients with hemifacial atrophy with 10 patients in each group [64]. They did not count the number of SVF cells used but the ratio between fat used for transfer and SVF isolation was 1:1. Results were evaluated by changes in volume estimated by CT scans up to 6 months postoperatively. A significantly higher survival rate was seen in the CAL group 68.3 versus 58.5 % in the regular lipotransfer group.

#### Randomized Studies

Koh et al. have conducted a study with 10 patients with Parry–Romberg disease who underwent lipotransfer to the face where 5 patients underwent CAL [65]. All patients had a primary and secondary lipotransfer (secondary 14 days after primary lipotransfer) and in the time period between these two procedures ASCs were cultured up to passage 3 and regardless of amount of fat that was transplanted 1 × 10^7^ ASCs were co-injected at the secondary procedure. Results were evaluated with CT scans and 3D photography after 6 months. The CAL group had undergone larger lipotransfers compared with the control group. The resorption level was still smaller in the CAL group at 20.59 versus 46.81 % in the control group, which was statistically significant. There are several pitfalls in this study as blinding and allocation of patients was not described. In addition the fat graft used was stored fat, but it was not described how the tissue was stored and the viability of the injected tissue was not examined.

Kølle et al. have conducted the so far only blinded randomized clinical trial comparing the survival of CAL enriched with autologous ASCs versus regular lipotransfer [66]. Ten patients completed the trial in which a bolus injection of fat was injected to the upper arm on each site with a total volume of 34 ml. On one side, the fat graft was enriched with 2.2 × 10^7^ ASCs/ml of fat and on the other side the fat graft was not enriched. The patients were followed for 121 days when the grafts were explanted for histologic analysis. MRI was performed preoperatively, immediately after lipotransfer and after 121 days. The survival rate for ASC-enriched grafts and control grafts, respectively, was 80.9 % (76.6–85.2) and 16.3 % (11.1–21.4) of the initial volumes (*p* < 0.0001) yielding a relative survival improvement of 5.0. Histologic analysis revealed a significantly higher quality of the remaining graft but interestingly the capillary density was not increased as most preclinical studies have shown. Although the strength of this study was the possibility to explant the graft for histologic analysis, it is also the downfall, as the injection method using a bolus injection instead of opting for the micro-droplet injection technique, surely has affected survival of the graft.

## Discussion

In this review, we have presented the current evidence for the efficacy of CAL. In preclinical studies, both SVF and ASCs seem to provide evidence for the superiority of CAL. The clinical studies using SVF are not as well designed as studies using ASCs but they show promising results.

Despite the many published studies so far it is still unknown, how many cells are needed for the optimal survival rate and if it is even worth sacrificing lipoaspirate for stem cell isolation when this could have been used directly or at a later time point for additional lipotransfer. This question is very important when using SVF because a large volume is needed to obtain meaningful cell enrichment. When using ASCs this is less important as cells can be culture-expanded; however, this also has the pitfall of prolonging the treatment to a two-stage procedure. It is apparent that although no clear-cut conclusion can be made regarding using SVF or ASC, they both have their strengths and limitations when taking a practical, legal, and logistic approach. The pitfalls of both cell populations beg the question of whether the same results could be obtained by performing two simple lipotransfers instead. This is especially true if your regular technique results in more than 70 % survival rate, because this leaves very little room for improvement with CAL. The survival rate of the regular lipotransfer in the human studies varied from 16.3 to 79 % and this impacts the window of opportunity for CAL to improve the treatment. The only properly design randomized clinical trial using a bolus technique resulted in only 16.3 % survival of regular lipotransfer and it would have been very interesting to see the results of a similar study using a more conventional injection technique.

The quality of reporting outcomes in preclinical studies should be standardized so that they always include a volumetric/weight retention analysis and morphologic characterization including vessel density of the retained fat as lipotransfer is not only about volume but also quality of the volume. In the same manner, clinical studies should all include an objective volumetric assessment of CAL using either CT or MRI before and after surgery.

Another crucial aspect of CAL is the safety of the technique. Several in vitro and in vivo experimental studies have suggested that SVF and ASCs can support tumor growth through paracrine mechanisms [67–69]. The concern is perhaps not so much that CAL or even just regular lipotransfer can act tumorigenic but rather can support and promote the growth of cancer cells if any are left behind after primary treatment. Lipotransfer is especially useful for breast reconstruction following cancer. Especially in cases where breast-conserving surgery has been performed, it seems reasonable to wait a few years after surgery before performing lipotransfer. More studies are needed to examine if CAL and lipotransfer are correlated with increased cancer recurrence risk in relevant patient populations [70, 71].

## Future Perspectives

For soft tissue reconstruction, CAL is at a crucial time point where the transition from very exciting preclinical results to clinical therapy is in process. The published human studies so far show promising results, and further properly designed clinical trials are needed in relevant patient groups to establish in which cases this technique could be relevant and superior to two separate regular lipotransfers.
